# Catalyst-free synthesis of tetrahydrodipyrazolopyridines via an one-pot tandem and green pseudo-six-component reaction in water

**DOI:** 10.1186/s13065-022-00802-4

**Published:** 2022-03-04

**Authors:** Mina Keihanfar, Bi Bi Fatemeh Mirjalili

**Affiliations:** Department of Chemistry, College of Science, Yazd University, Yazd, Iran

**Keywords:** Tetrahydrodipyrazolopyridines (THDPP’s), Multicomponent reaction, Environmentally friendly protocol, Catalyst-free reaction

## Abstract

**Background:**

A new, green and environmentally friendly protocol has been developed for the synthesis of tetrahydrodipyrazolopyridine derivatives. The structures of these products were determined in terms of melting point, FTIR, NMR and Mass spectroscopy.

**Results:**

The tetrahydrodipyrazolopyridine derivatives were synthesized in water through a catalyst-free pseudo-six-component reaction of hydrazine hydrate, ethyl acetoacetate, ammonium acetate and aldehyde at room temperature.

**Conclusions:**

This novel procedure has some advantages such as aqueous media, high yield and simple work-up.

**Graphical Abstract:**

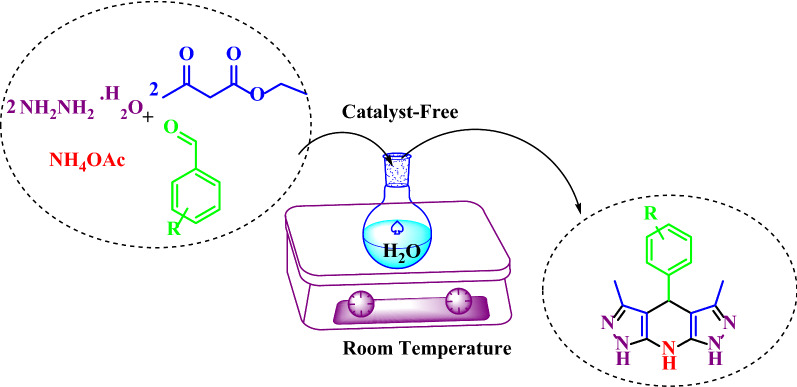

**Supplementary Information:**

The online version contains supplementary material available at 10.1186/s13065-022-00802-4.

## Introduction

**Fig. 1:**
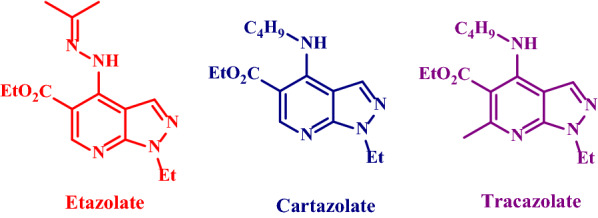
Pyrazolopyridine with pharmaceutical activity

Multicomponent reactions (MCRs) are selective, simple and effective as compared to the conventional multistep synthesis [1–4]. They have been utilized to reduce environmental pollution. Water, as a green solvent, is presumed to speed up some organic reactions through hydrophobic effects [5]. Therefore, catalyst-free organic reactions in water that yield resolvable products are attractive for many organic chemists [6, 7]. THDPPs fuse the heterocyclic moieties of pyrazolopyridine with pharmaceutical activities, which exist in Etazolate, Cartazolate and Tracazolate drugs [8] (Fig. 1).

Meanwhile, Due to the existence of two biologically active moieties, namely pyrazole and 1,4-dihydropyridine pyrazole in the structure of THDPPs, these compounds have various pharmaceutical applications such as antiallergic, anti-herpetic and anxiolytic effects [9, 10], anti-Leishmania activities [11] and HIF 1α-prolyl hydroxylase inhibition [12].

A common protocol for the synthesis of THDPP is the reaction of aldehydes, hydrazine hydrate, ethyl acetoacetate, and ammonium acetate in a multi-component manner with the presence of a catalyst. Previously, pseudopolymeric magnetic nanoparticles [13], KCC-1-NH_2_-DPA [14], CuFe_2_O_4_@HNTs [15], acetic acid [16], and carbonaceous material (CSO_3_H) [17], Fe_3_O_4_/KCC1/IL/HPWMNPs [24], Nano-CdZr_4_(PO_4_)_6_ [25], Nano-Fe_3_O_4_@SiO_2_–SO_3_H [23], FeNi_3_-ILs MNPs [21], Nano-CuCr_2_O_4_ [26], Nano-ovalbumin [20], M(II)/Schiff base@MWCNT-Fe_3_O_4_/SiO_2_ [27]. Meanwhile, a catalyst free protocol using ammonium carbonate instead of ammonium acetate has been reported [16], Ammonium carbonate is a basic salt and can catalysis the synthesis of THDPP’s. In here, we have used ammonium acetate as neutral salt for production of ammonia in water and absence of any catalyst.

**Scheme 1:**
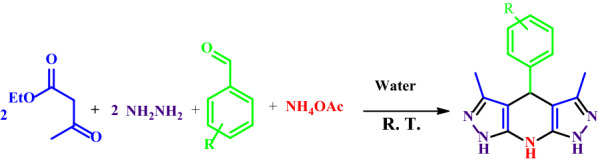
Synthesis of THDPP’s in water

In this study, THDPPs are synthesized with a green catalyst-free protocol implemented in a water medium at room temperature (Scheme 1).

## Experimental

### Materials and methods

#### General

A Bruker, Equinox 55 spectrometer was used to record the Fourier transforms infrared (FT-IR) spectra. A Bruker (DRX-400 Avance) nuclear magnetic resonance (NMR) instrument was also used to record the NMR spectra. In addition, a Buchi B-540 B.V.CHI apparatus served to determine the melting points of the compounds. Mass spectrometry spectra were recorded with a Agilent Technology (HP), Model: 5793, Ion source: Electron Impact (EI), 20-EV, 230 °C, and Quadrupole analyzer.

### Chemistry

#### General procedure for the synthesis of THDPP’s

Firstly, a solution of 2.0 mmol of hydrazine hydrate, 2.0 mmol of ethyl acetoacetate and 3 mL of water was stirred in a 25-mL round-bottom flask at room temperature. Secondly, 1.0 mmol of aldehyde and 4.0 mmol of ammonium acetate were added to it and stirred at room temperature. The reaction was monitored by thin-layer chromatography (TLC; *n*-hexane:EtOAc, 70:30). After the completion of the reaction, the solution was diluted with cold water, and the product was appeared as water insoluble solid which isolated by simple filtration.

## Results and discussion

Although, catalytic synthesis of THDPPs protocols have many advantages such as high yields of products and short reaction time. But the hard work-up and expensive catalysts are some of drawbacks of them. Therefore, we have decided to design a catalyst-free protocol for synthesis of THDPPs.

In order to optimize the reaction conditions for the preparation of THDPPs in the absence of a catalyst, some reactions were performed between ethylacetoacetate, ammonium acetate, 4-chlorobenzaldehyde and hydrazine hydrate in the presence of different solvents (Table 1). As the results indicated, tetrahydrodipyrazolopyridines could be synthesized in good-to-high yields and short reaction times.

**Table 1:** Optimization of the reaction conditions for the synthesis of 4-(4-chlorophenyl)-3,5-dimethyl-1,4,7,8-tetrahydrodipyrazolo[3,4-b:4′,3′-e]pyridine
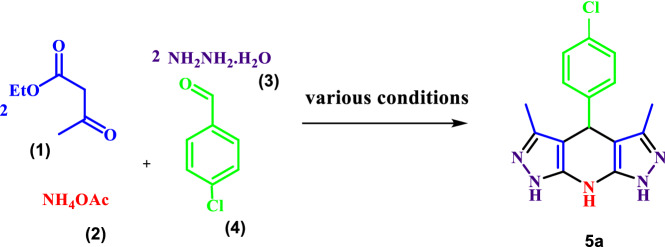

Entry	Solvent	Conditions^a^	Time (min)	Yield (%)^b^
1	EtOH	r.t	180	–
2	EtOH	Reflux	120	23
3	H_2_O	Reflux	60	53
4	**H**_**2**_**O**	**r.t**	**45**	**98 [This work]**

^a^The molar ratio of hydrazine hydrate (2 mmol), ethyl acetoacetate (2 mmol),4-chlorobenzaldehyde (1 mmol) and ammonium acetate (4 mmol) is equal to 2:2:1:4

^b^Isolated yields

Hydrazine and ammonium acetate are soluble in water, ethyl acetoacetate is partially soluble and aldehyde is insoluble in water. In the first step, hydrazine hydrate and ethyl acetoacetate react in water to produce a insoluble intermediate which react with aldehyde in water cage.

Regarding the conditions for the synthesis of THDPPs, the optimization process was implemented with different aldehydes, hydrazine hydrate, ethyl acetoacetate and ammonium acetate (Table 2).

**Table 2:** Synthesis of THDPP’s (5_a–m_) in water in room temperature
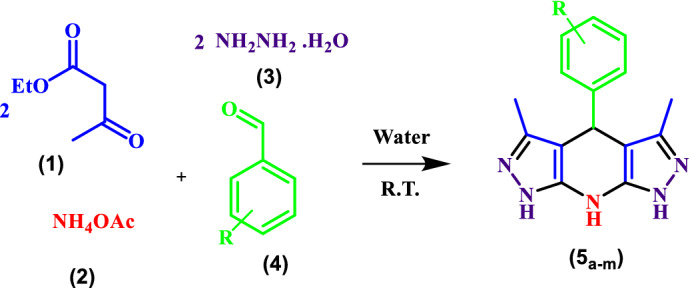

Entry	R	Product	Time (min)	Yield (%)^b^	M.P (°C)	Lit. M.P. (C) [Reference]
1	4-Cl	5a	45	98	244–246	254–256 [19]
2	4-NO_2_	5b	60	96	274–276	278–283 [19]
3	3-NO_2_	5c	50	98	268–270	282–284 [20]
4	4-OH	5d	80	96	266–268	267–268 [21]
5	4-Br	5e	75	96	222–224	221–224 [19]
6	2-Cl	5f	105	94	162–164	164–165 [22]
7	4-N(CH_3_)_2_	5g	90	85	238–239	240–242 [19]
8	4-Me	5h	180	95	240–242	241–243 [20]
9	4-F	5i	75	97	255–257	258–260 [18]
10	4-OCH_3_	5j	90	98	187–189	188–190 [20]
11	3-OMe-4-OH	5k	90	98	256–258	256–258 [20]
12	3,4-(OH)_2_	5l	90	83	208–210	208–210 [20]
13	4-CHO	5 m	120	91	> 300°	> 300° [23]

Reaction conditions: hydrazine hydrate (2 mmol), ethyl acetoacetate (2 mmol), 4-chloro benzaldehyde (1 mmol) and ammonium acetate (4 mmol), water (3 mL) at room temperature

**Scheme 2:**
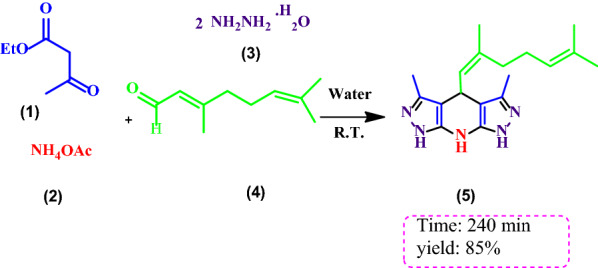
Synthesis of 4-(Z)-2,6-dimethylhepta-1,5-dien-1-yl)-3,5-dimethyl-1,4,7,8-tetrahydrodipyrazolo[3,4-b:4′,3′-e]pyridine

Meanwhile, we have synthesis THDPP using citral as aliphatic aldehyde (Scheme 2).

**Scheme 3:**
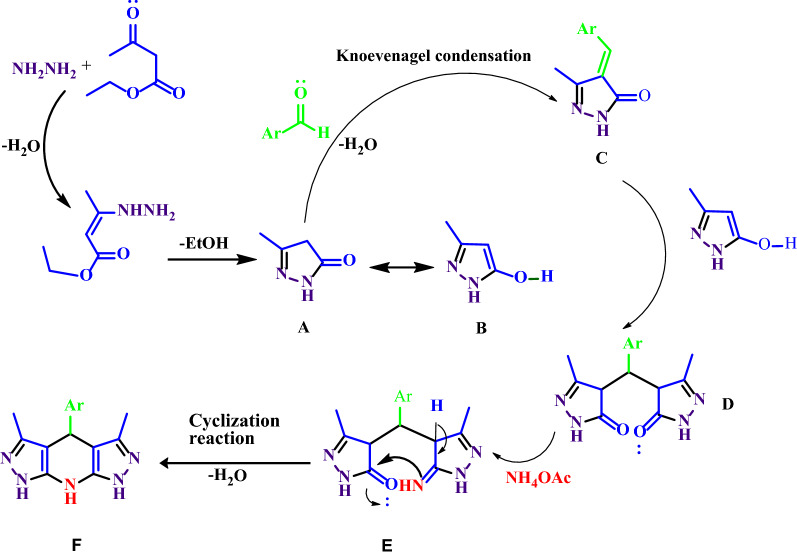
A proposed mechanism for synthesis of THDPP’s

A mechanism proposed for the catalyst-free synthesis of THDPPs is shown in Scheme 3. Ammonium acetate was used as a source of nitrogen for the formation of 1,4-dihydropyridine ring in the THDPPs. Hydrazine is soluble and ethylacetoacetate is partially soluble (2.86 g/100 mL) in water. Initially, the reaction was begun with the versatile condensation of ethyl acetoacetate with hydrazine and then the elimination of ethanol to form pyrazolone A (B, tautomer of A) as a water insoluble solid. The A (B) and aldehyde are hydrophobia materials and react in a water cage to form the intermediate C through the Knoevenagel condensation. Then, the second molecule of B was condensed with C via Michael reaction to produce bipyrazolone D. Ammonia, which was produced from ammonium acetate, condensed with D to form imine E which produced product F through intramolecular cyclization, tautomerization and water removal.

In order to show the superiority of the THDPPs synthesis process in the absence of catalysts, this process was compared to some others in terms of conditions, reaction time and yield. The results are listed in Table 3.

**Table 3:** The comparison of catalyst-free protocol with other methods for synthesis of 5a
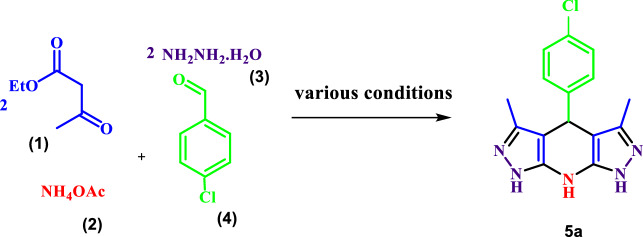

Entry	Catalyst	Conditions	Time (min)/yield^a^ (%) [Reference]
1	Fe_3_O_4_/KCC1/IL/HPWMNPs (0.0001 mg)	H_2_O/r.t	30/96 [24]
2	Nano-CdZr_4_(PO_4_)_6_ (0.6 mol%)	EtOH/reflux	43/88 [25]
3	Nano-Fe_3_O_4_@SiO_2_-SO_3_H (0.004 g)	EtOH/MW	20/90 [23]
4	FeNi_3_-ILs MNPs (0.002 g)	EtOH/reflux	48/86 [21]
5	Nano-CuCr_2_O_4_ (4 mol%)	EtOH/25 °C	50/90 [26]
6	Nano-ovalbumin (0.05 g)	H_2_O/55 °C	45/93 [20]
7	M (II)/Schiff base@MWCNT-Fe_3_O_4_/SiO_2_ (0.02 g)	–/r.t	90/85 [27]
8	Pseudopolymeric magnetic nanoparticles (10 mg)	EtOH/r.t	10–180/45–92 [13]
9	KCC-1-NH_2_-DPA (0.1 g)	EtOH, reflux	30/95 [14]
10	CuFe_2_O_4_@HNTs (5 mg)	EtOH, r.t	20/90–96 [15]
11	acetic acid	AcOH/reflux	300/90 [16]
12	carbonaceous material (CSO_3_H) (10 mg)	H_2_O/60 °C	360/86 [17]
13	Catalyst-free	H_2_O/r.t	45/98 [This work]

^a^Isolated yields

## Conclusions

In this study, an environmentally friendly protocol is introduced for the synthesis of THDPPs in a neutral aqueous medium without using any catalyst or organic solvent. In this green versatile protocol hydrophobia intermediate and aldehyde had been reacted under high pressure condition in water cage. In solubility of products in water caused having a simple work-up and purification of them. The attractive advantages of the protocol are excellent yields, mild reaction conditions, less pollution, short time reaction, simple workup and high-purity products.

## Supplementary Information

**Additional file 1:** Spectroscopic data for the synthesized tetrahydrodipyrazolo[3,4-b:4′,3′-e] pyridine derivatives.

## Data Availability

All data generated or analyzed during this study are included in this published article.

## Abbreviations

THDPP’s: Tetrahydrodipyrazolopyridines
MCRs: Multi-component reactions
FT-IR: Fourier transform infrared
NMR: Nuclear magnetic resonance
TLC: Thin layer chromatography
