# Supplementary material for: Catalyst-free synthesis of tetrahydrodipyrazolopyridines via an one-pot tandem and green pseudo-six-component reaction in water
Source: BMC Chem. 2022 Mar 4;16(1):9. doi: 10.1186/s13065-022-00802-4 (PMC8897970; doi:10.1186/s13065-022-00802-4)

**Catalyst-free Synthesis of Tetrahydrodipyrazolopyridines *via* an One-pot Tandem and Green Pseudo-Six-Component Reaction in Water**

Mina Keihanfar, Bi Bi Fatemeh Mirjalili*

^1^Department of Chemistry, College of Science, Yazd University, Yazd, Iran

Email:fmirjalili@yazd.ac.ir Telephone: +983531232672, Fax: +98 3538210644

**4-(4-Chlorophenyl)-3,5-dimethyl-1,4,7,8-tetrahydrodipyrazolo[3,4-*b*:4',3'-*e*]pyridine**

Cream solid, M.P. 244-246 °C, FT-IR (ATR) ῡ (cm^-1^): 3049, 2970, 1599, 1488, 1440, 1175, 1089, 863

^1^H NMR (400 MHz, DMSO-d_6_) /δ ppm: 11.33 (s, NH, 3H), 7.28 (d, *J*=8.4 Hz, 2H), 7.13 (d, *J*=8.4 Hz, 2H), 4.83 (s, 1H), 2.08 (s, 6H). C_15_H_14_ClN_5_, MS, m/z (%): 299.1 (M^+^), 220.1, 219, 185, 165.1, 128.1, 109.1, 98.1, 75.1.


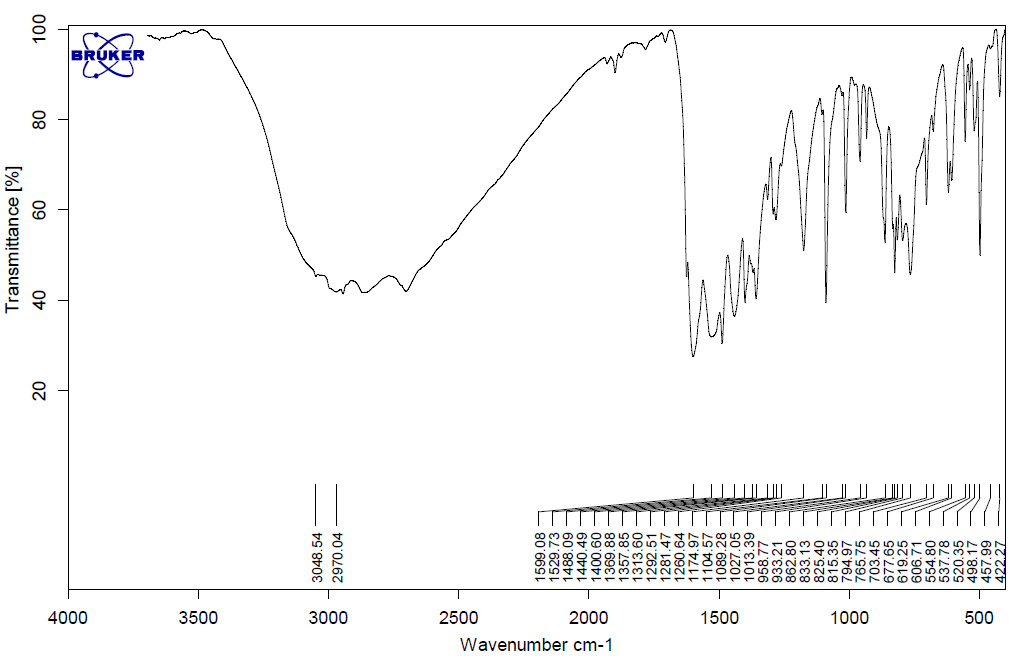


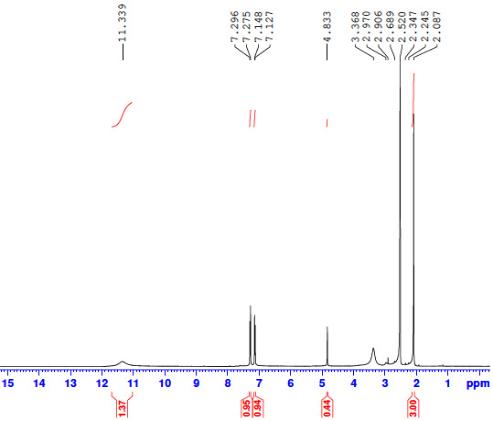


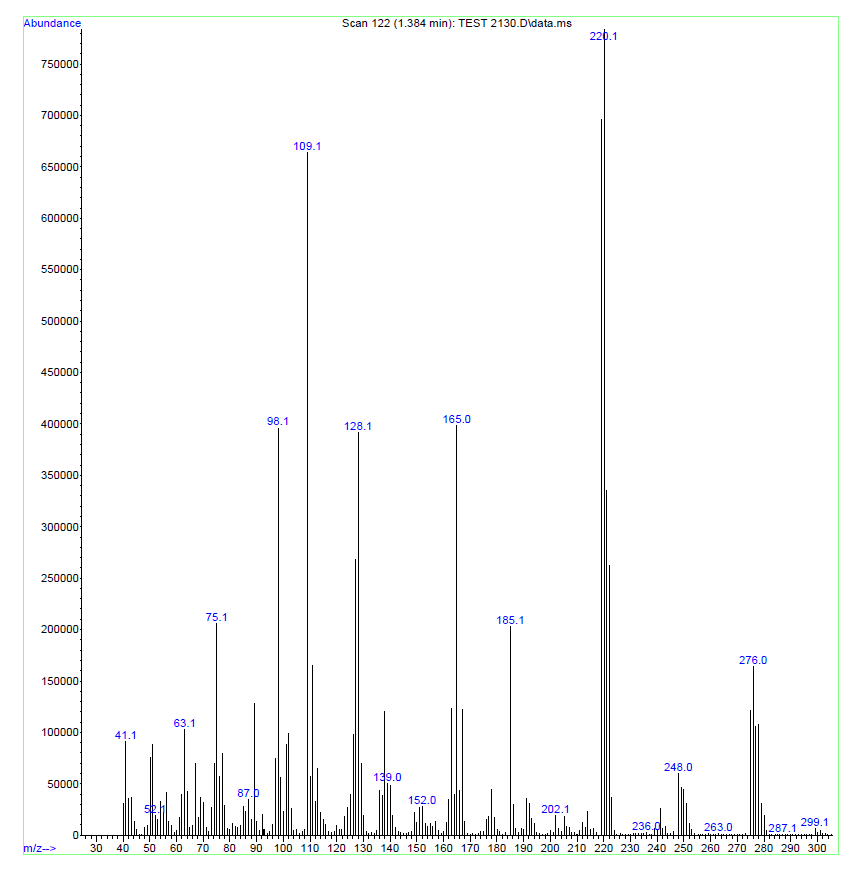


3,5-Dimethyl-4-(4-nitrophenyl)-1,4,7,8-tetrahydrodipyrazolo[3,4-*b*:4',3'-*e*]pyridine

Cream solid, M.P. 274-276 °C, FT-IR (ATR) ῡ (cm^-1^):3071, 1606, 1507, 1440, 1347, 857, 729

^1^H NMR (400 MHz, DMSO-d_6_) /δ ppm: 11.35 (s, NH, 3H), 8.13 (d, *J*=8.0 Hz, 2H), 7.39 (d, *J*=8.0 Hz, 2H), 4.99 (s, 1H), 2.10 (s, 6H). C_15_H_14_N_6_O_2_, MS, m/z (%): 311.2 (M^+^), 231.1, 202.1, 201, 185,184, 145.1, 127.1, 109.1, 98.1.

.


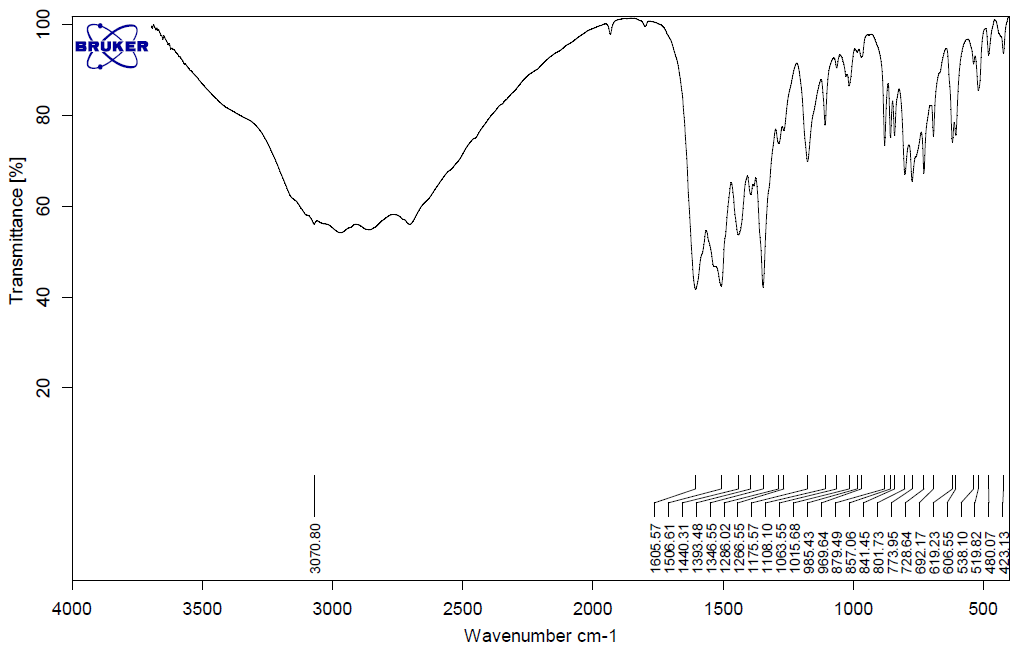


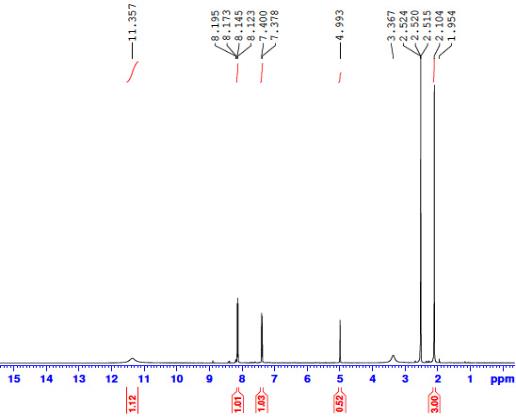


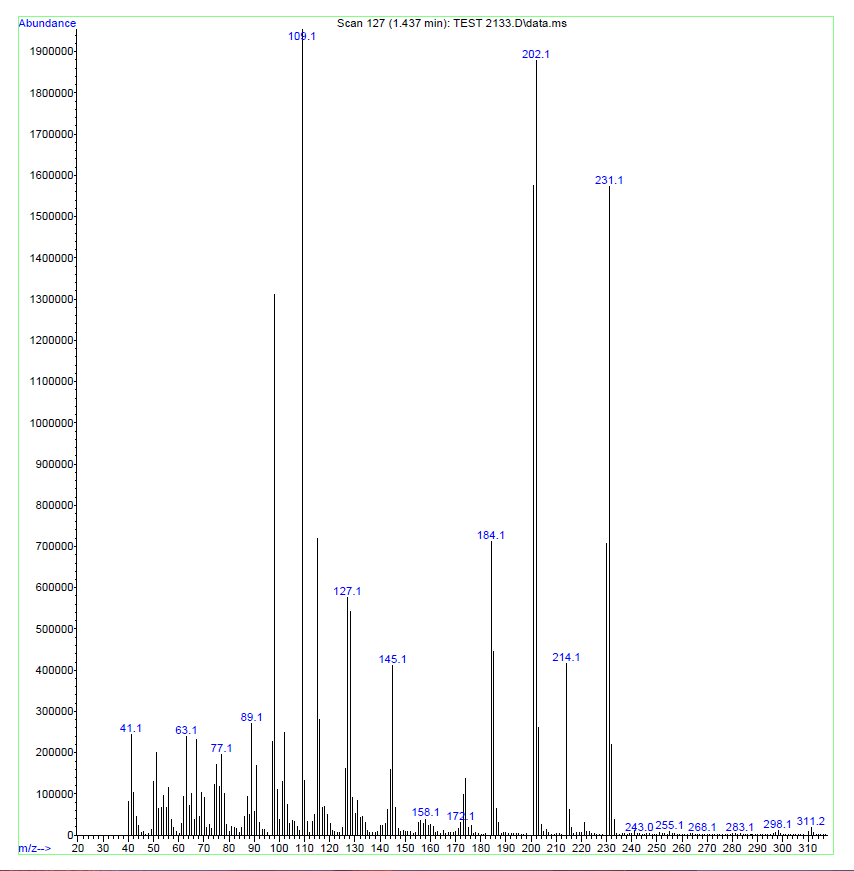


3,5-Dimethyl-4-(3-nitrophenyl)-1,4,7,8-tetrahydrodipyrazolo[3,4-*b*:4',3'-*e*]pyridine

Cream solid, M.P. 268-270 °C, FT-IR (ATR) ῡ (cm^-1^):3096, 1599, 1528, 1440, 1344, 1180, 899, 798, 762, 690

^1^H NMR (400 MHz, DMSO-d_6_) /δ ppm: 11.41 (s, NH, 3H), 8.04 (d, *J*=7.6 Hz, 1H), 7.97 (s, 1H), 7.59 (t, *J*=7.6 Hz, 1H), 7.54 (d, *J*=8.0 Hz, 1H), 5.01 (s, 1H), 2.10 (s, 6H). C_15_H_14_N_6_O_2_, MS, m/z (%): 311 (M^+^), 231.1, 202.1, 184, 128, 109.1, 80, 64, 48.


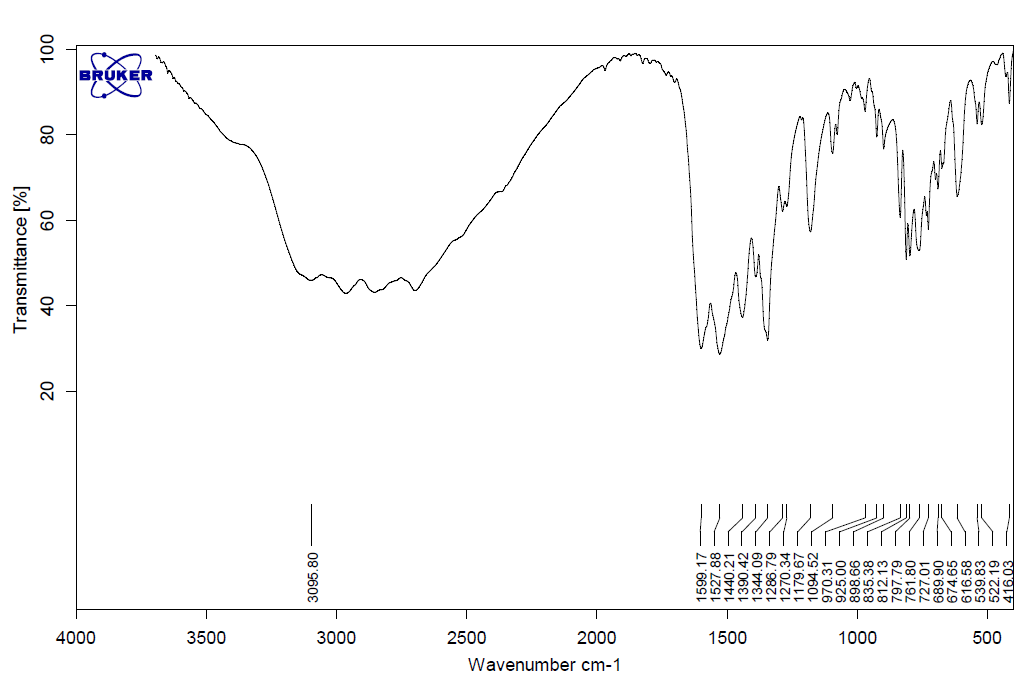


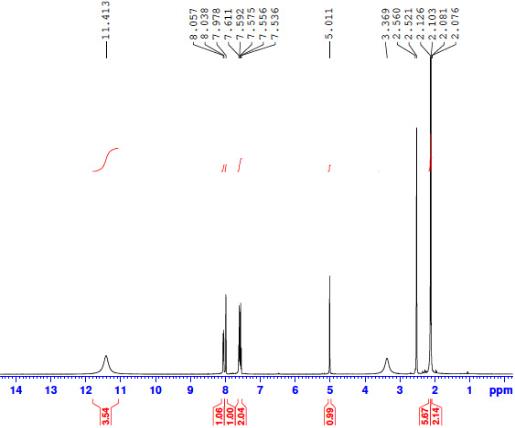


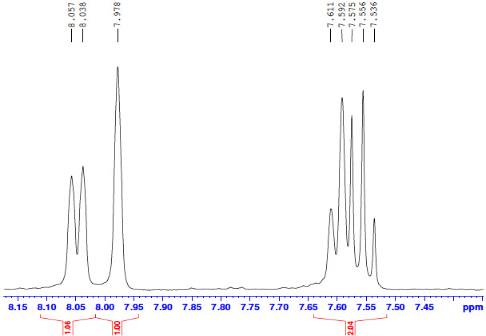


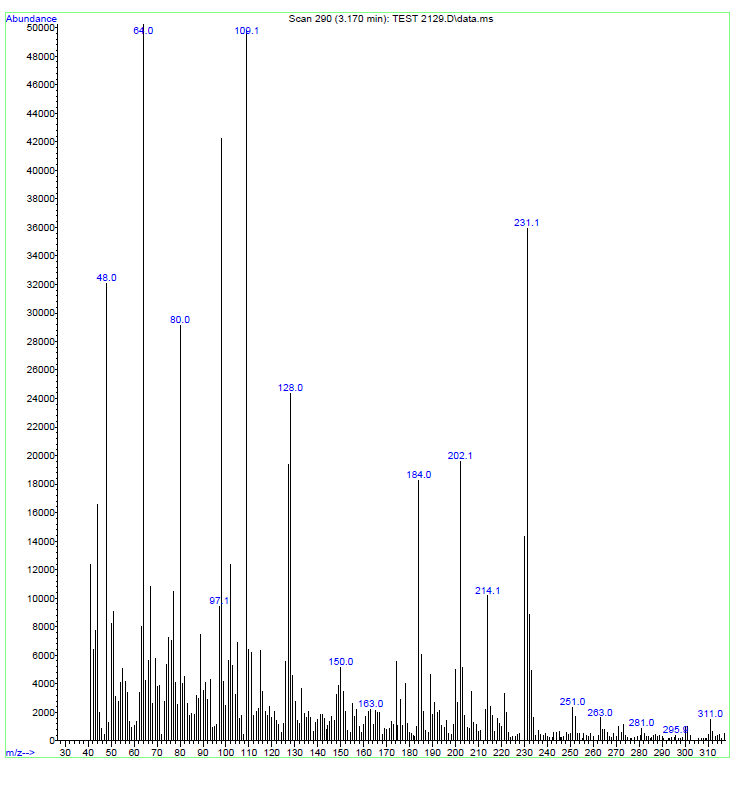


3,5-Dimethyl-4-(4-hydroxyphenyl)-1,4,7,8-tetrahydrodipyrazolo[3,4-*b*:4',3'-*e*]pyridine

White solid, M.P. 266-268 °C, FT-IR (ATR) ῡ (cm^-1^):3267, 1616, 1561, 1514, 1467, 1238, 1116, 1068, 844

^1^H NMR (400 MHz, DMSO-d_6_) /δ ppm: 11.30 (s, NH, 3H), 9.08 (s, OH, 1H), 6.92 (d, *J*=8.4 Hz, 2H), 6.60 (d, *J*=8.4 Hz, 2H), 4.72 (s, 1H), 2.10 (s, 6H). C_15_H_15_N_5_O, MS, m/z (%): 283.1 (M^+^), 202.1, 185.1, 145.1, 127.1, 109.1, 98.1.


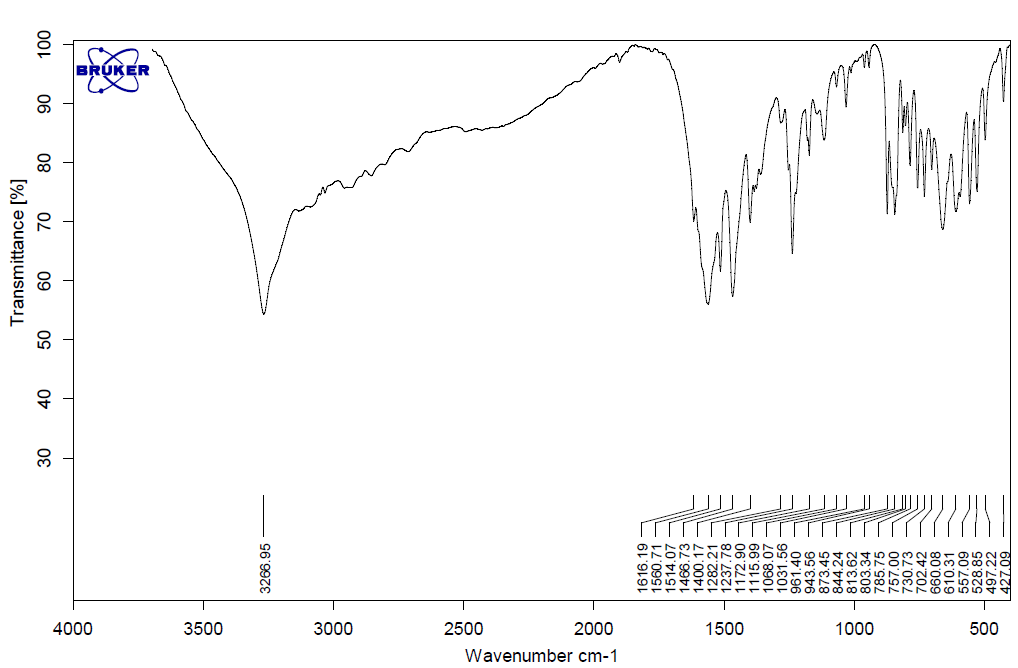

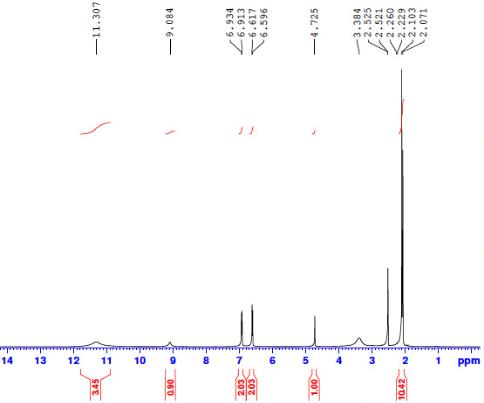


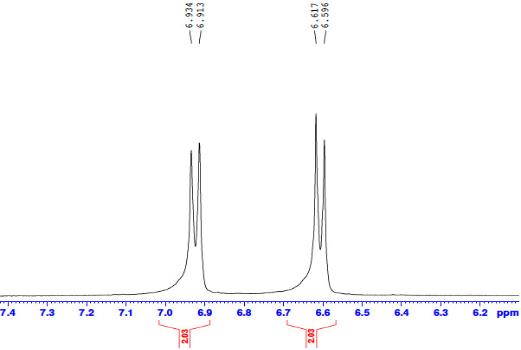


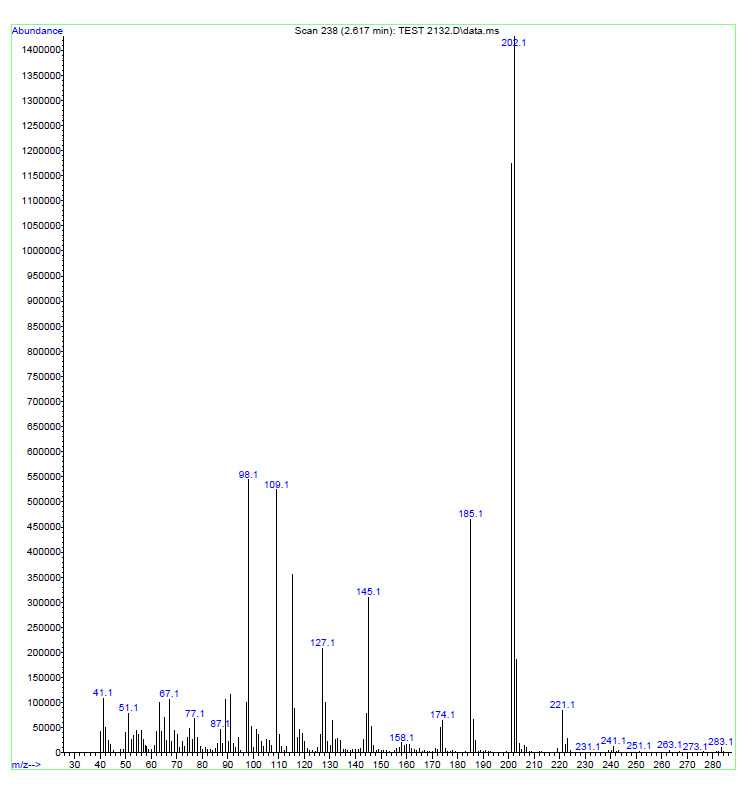


**4-(2-Chlorophenyl)-3,5-dimethyl-1,4,7,8-tetrahydrodipyrazolo[3,4-*b*:4',3'-*e*]pyridine**

Yellowish white solid, M.P. 162-164 °C, FT-IR (KBr) ῡ (cm^-1^):3069, 1616, 1541, 1439, 750

^1^H NMR (400 MHz, DMSO-d_6_) /δ ppm: 11.09 (s, NH, 3H), 7.56 (d, *J*=7.2 Hz, 1H), 7.34 (dd, *J*=8.0 Hz, *J*=1.2 Hz, 1H), 7.24 (t, *J*=7.6 Hz, 1H), 7.18 (td, *J*=7.6 Hz, *J*=1.2 Hz, 1H), 5.11 (s, 1H), 2.10 (s, 3H), 1.95 (s, 3H). C_15_H_14_ClN_5_, MS, m/z (%): 299 (M^+^), 220.1, 206, 185.1, 165.1, 128.1, 109, 98.1, 75.1.


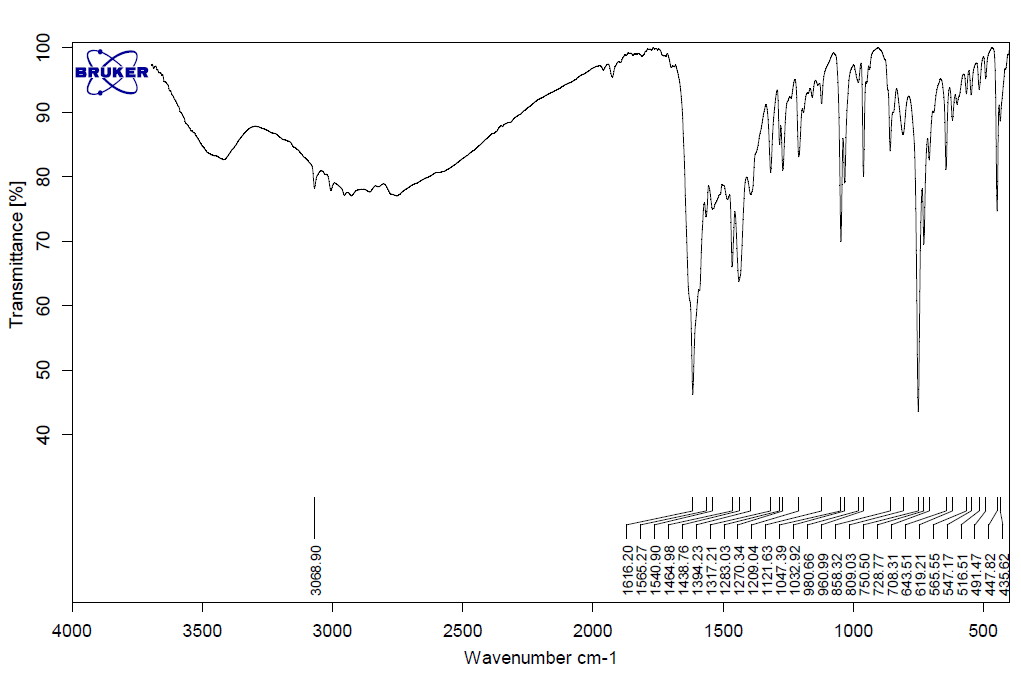


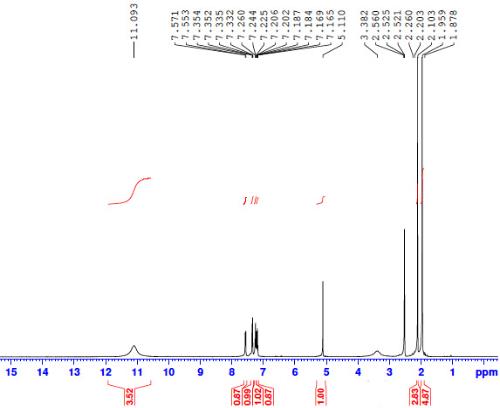


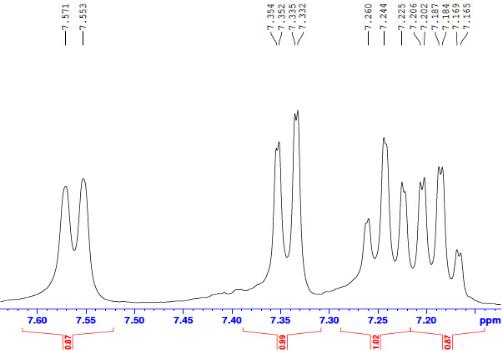


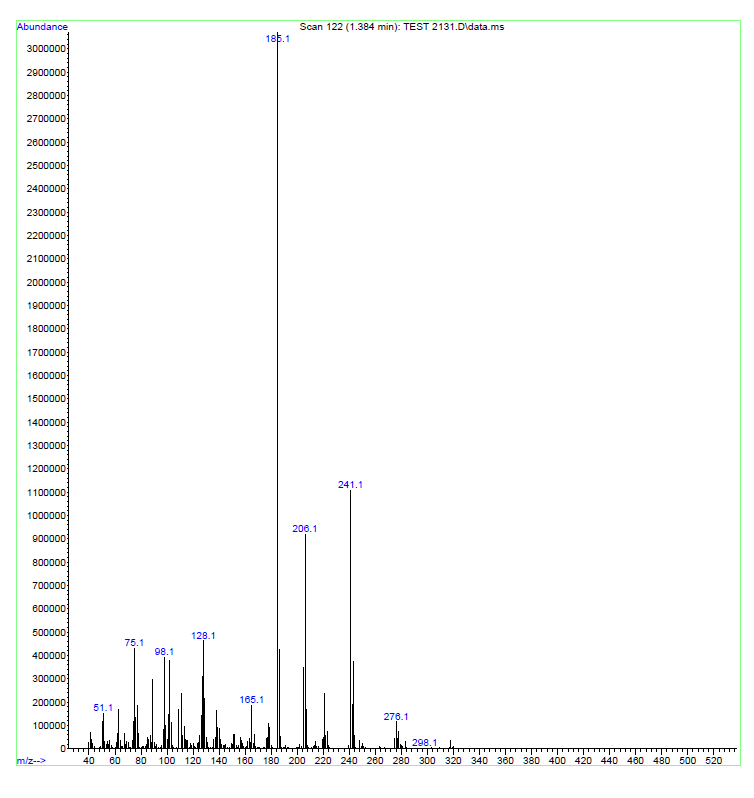


**3,5-Dimethyl-4-(*p*-tolyl)-1,4,7,8-tetrahydrodipyrazolo[3,4-*b*:4',3'-*e*]pyridine**

Yellowish white solid, M.P. 240-242 °C, FT-IR (ATR) ῡ (cm^-1^):3301, 1608, 1510, 1450, 1412, 818, 749

^1^H NMR (400 MHz, DMSO-d_6_) /δ ppm: 11.39 (s, NH, 3H), 7.02 (s, 4H), 4.74 (s, 1H), 2.24 (s, 3H), 2.15 (s, 3H), 2.10 (s, 3H). C_16_H_17_N_5_, MS m/z (%): 280.1 (M^+^), 202.1, 185.1, 145.1, 128.1, 109.1, 98.1, 80, 64, 48.


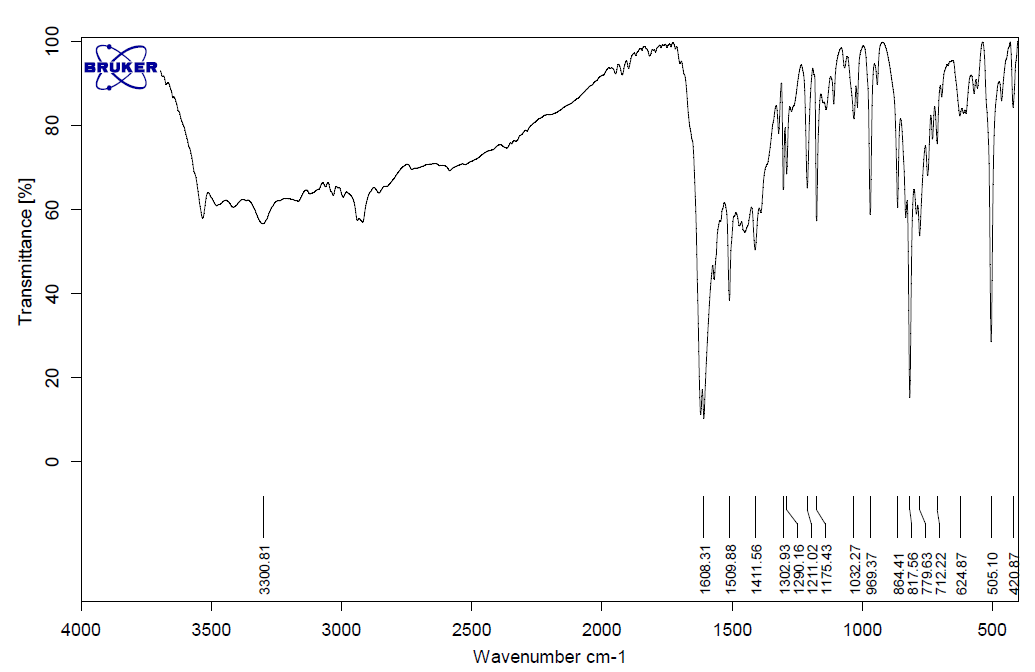


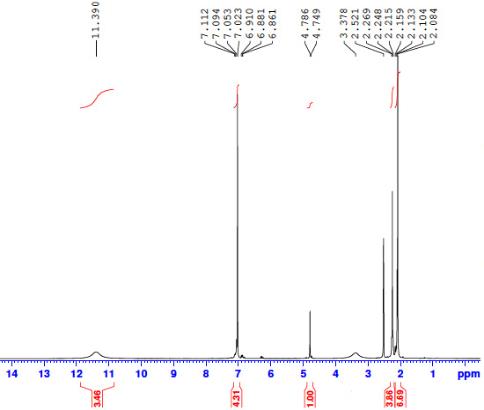


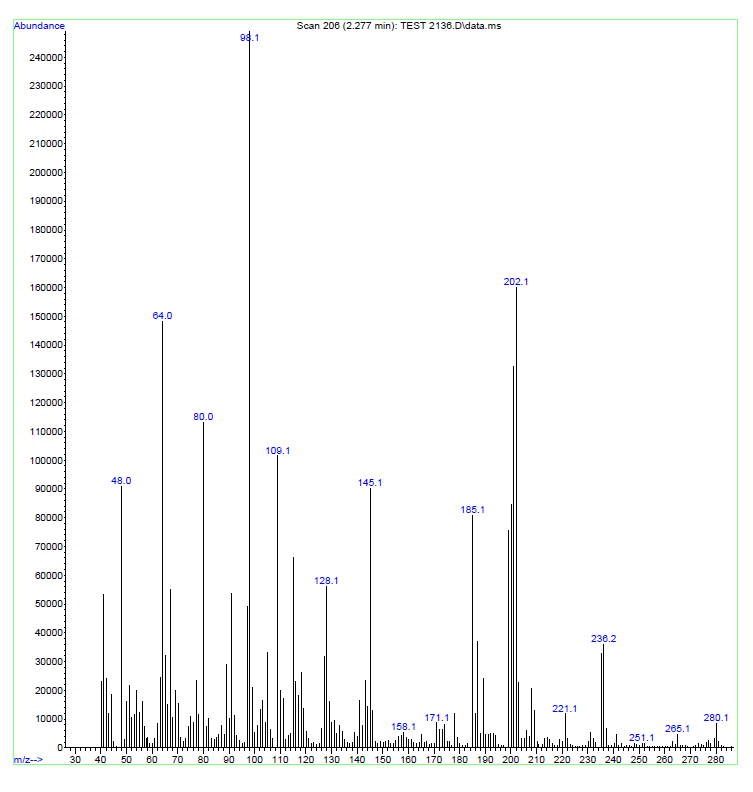


**4-(4-Fluorophenyl)-3,5-dimethyl-1,4,7,8-tetrahydrodipyrazolo[3,4-*b*:4',3'-*e*]pyridine**

White solid, M.P. 255-257 °C, FT-IR (KBr) ῡ (cm^-1^):3048, 2968, 1598, 1507, 1441, 1222, 830, 760

^1^H NMR (400 MHz, DMSO-d_6_) /δ ppm: 11.36 (s, NH, 3H), 7.15 (dd, *J*=8.0 Hz, *J*=5.6 Hz, 2H), 7.04 (t, *J*=8.8 Hz, 2H), 4.83 (s, 1H), 2.09 (s, 6H). C_15_H_14_FN_5_, MS, m/z (%): 282.1 (M^+^), 244.1, 216.1, 149.1, 122.1, 109.1, 95.1, 75.1, 64, 48.


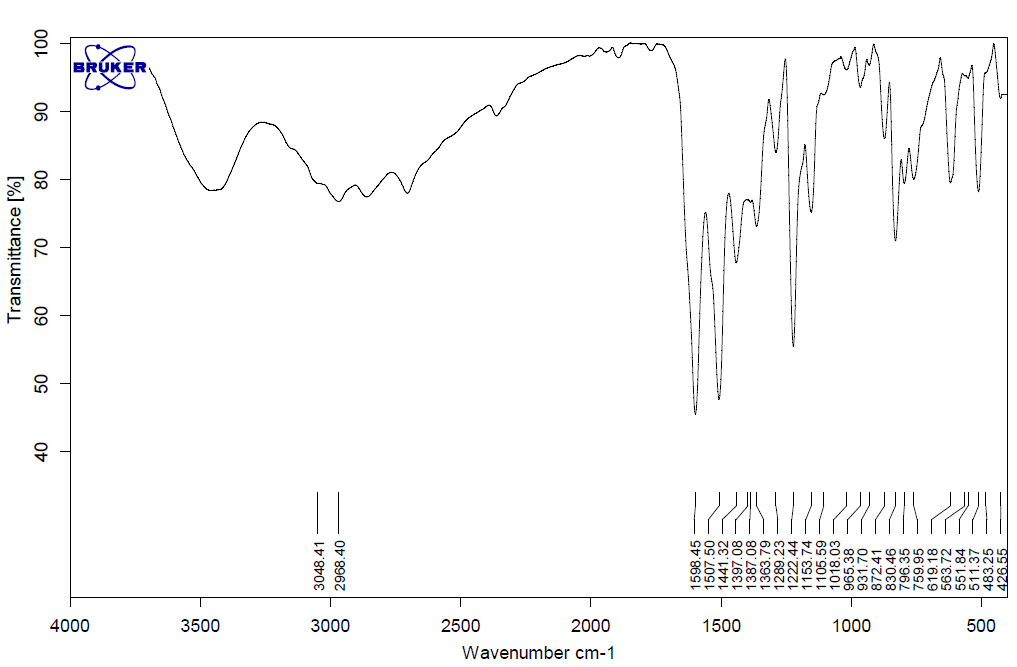


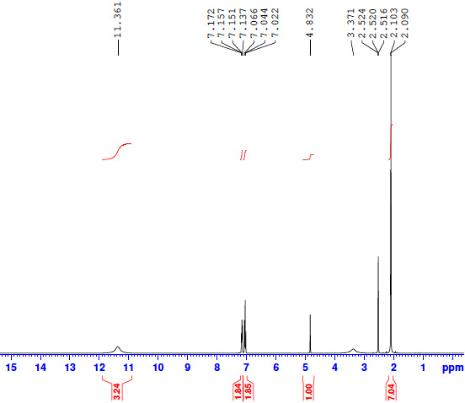


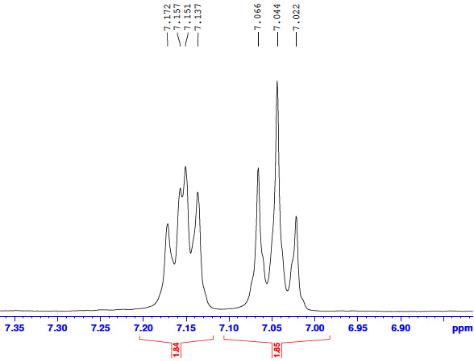

**
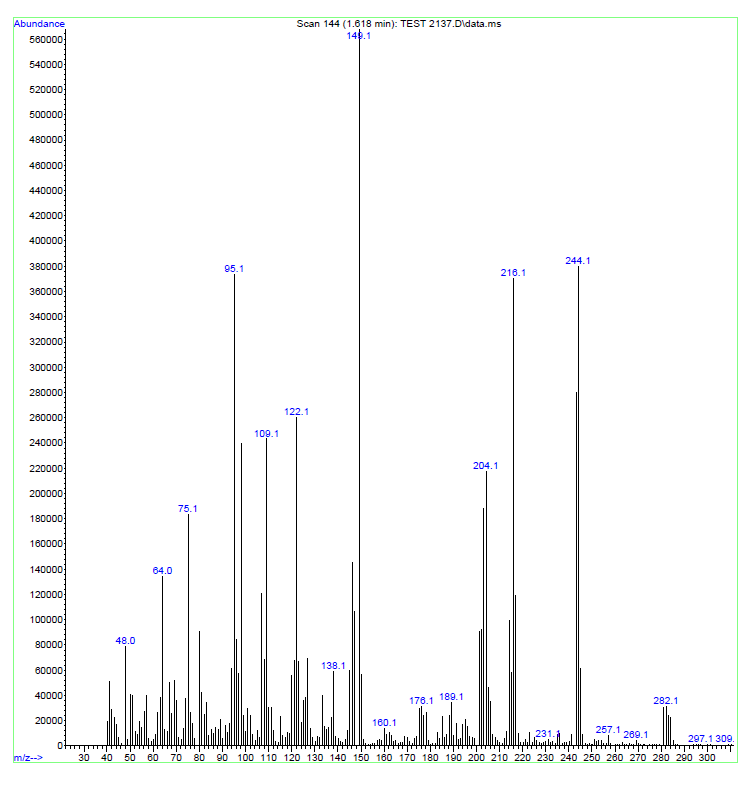
**

**4-(4-Methoxyphenyl)-3,5-dimethyl-1,4,7,8-tetrahydrodipyrazolo[3,4-*b*:4',3'-*e*]pyridine**

Cream solid, M.P. 245-247 °C FT-IR (ATR) ῡ (cm^-1^): 3269, 3183, 1599, 1508, 1469, 1247, 1022, 830

^1^H NMR (400 MHz, DMSO-d_6_) /δ ppm: 11.14 (s, NH, 3H), 7.04 (d, *J*=8.0 Hz, 2H), 6.78 (d, *J*=8.0 Hz, 2H), 4.77 (s, 1H), 3.70 (s, 3H), 2.10 (s, 6H). C_16_H_17_N_5_O, MS m/z (%): 296.1 (M^+^), 268.1, 216.1, 202.1, 201, 161.1, 109.1, 98.1, 64, 41.1.


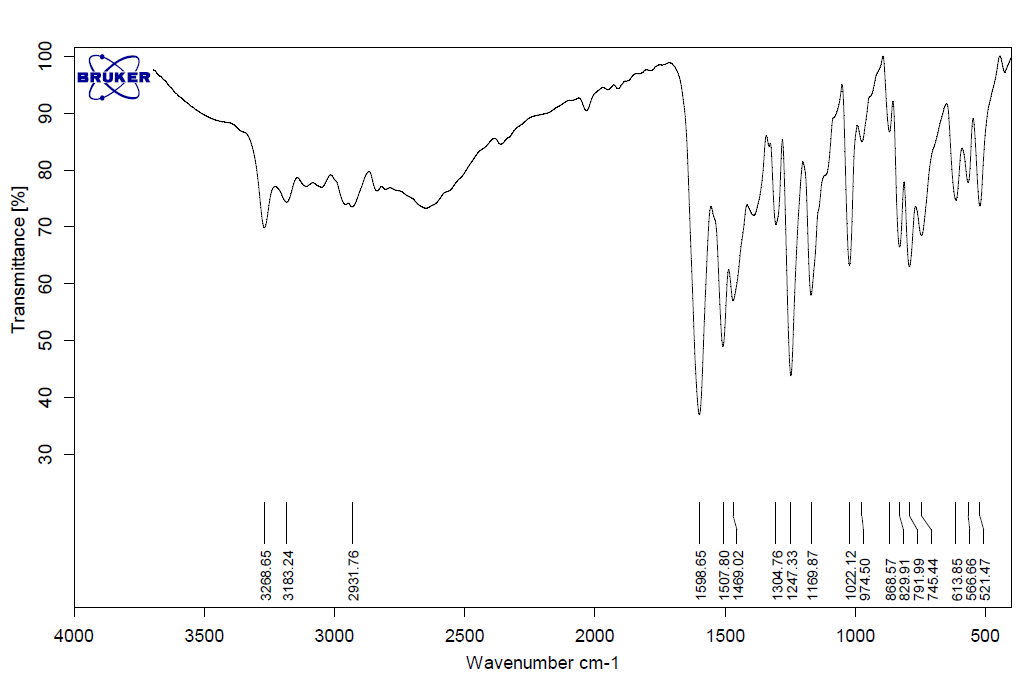


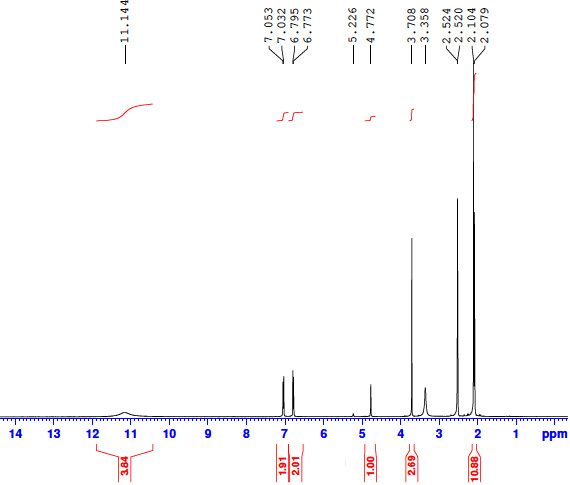


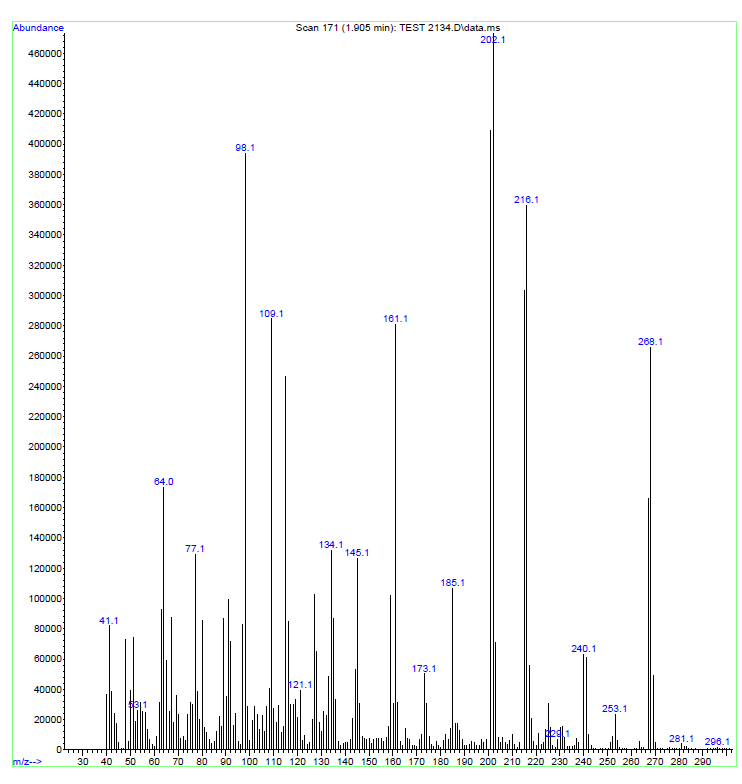


**4-(4-Hydroxy-3-methoxyphenyl)-3,5-dimethyl-1,4,7,8-tetrahydrodipyrazolo[3,4-*b*:4',3'-*e*]pyridine.**

White solid, M.P. 256-258°C, FT-IR (ATR) ῡ (cm^-1^):3373, 3195, 1609, 1533, 1485, 1260, 1130, 1038, 861, 844, 788,^1^H NMR (400 MHz, DMSO-d_6_) /δ ppm: 11.29 (s, NH, 3H), 8.67 (s, OH, 1H), 6.75 (d, *J*=1.6 Hz, 1H), 6.62 (d, *J*=8.4 Hz, 1H), 6.55 (dd, *J*=8.0 Hz, *J*=1.2 Hz, 1H), 4.74 (s, 1H), 3.65 (s, 3H), 2.06 (s, 6H). C_16_H_17_N_5_O_2_, MS, m/z (%): 312.1 (M^+^), 202.1, 201, 185.1, 145.1, 109.1, 98.1, 64, 48.


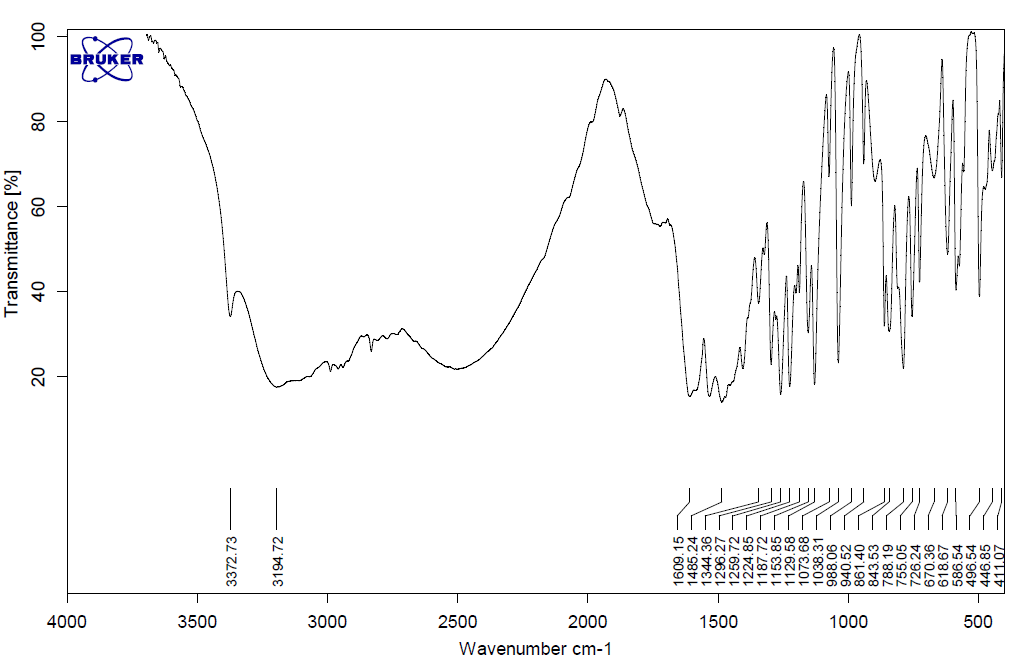


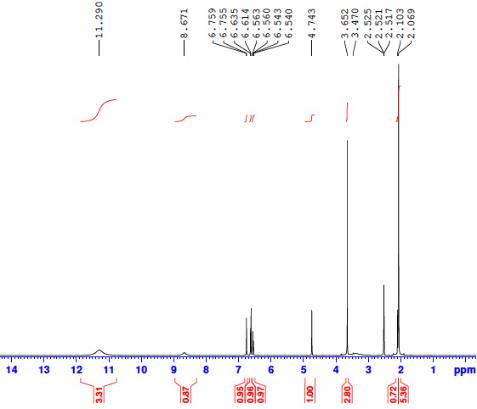


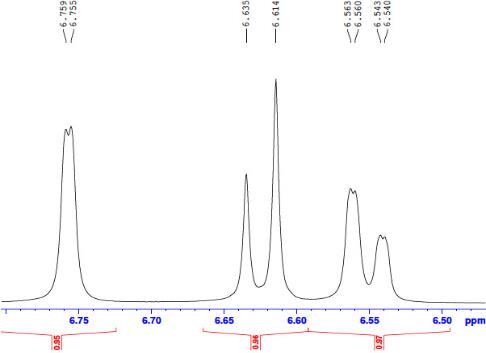


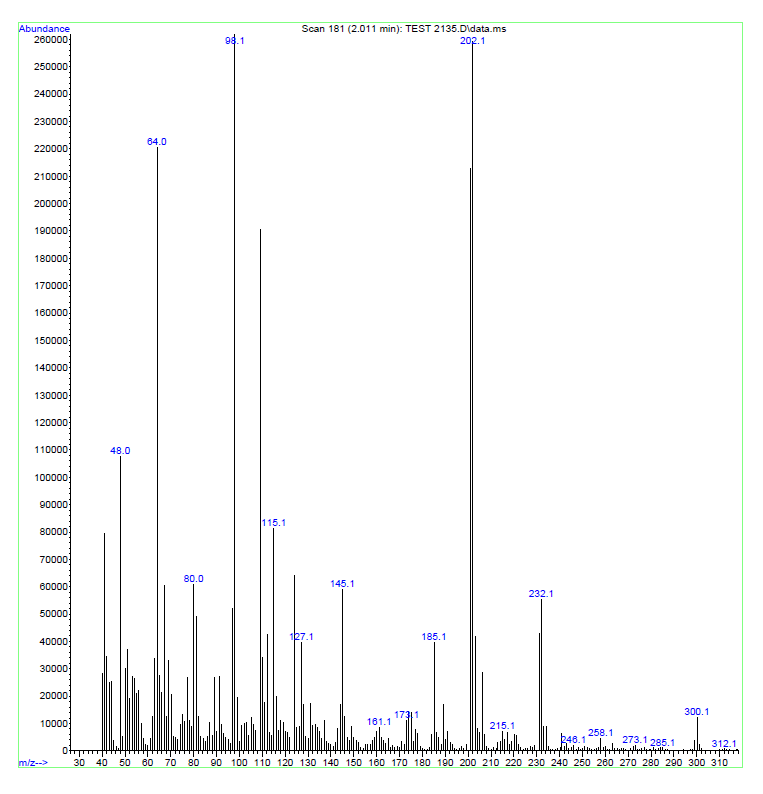


**4-(3,4-Dihydroxyphenyl)-3,5-dimethyl-1,4,7,8-tetrahydrodipyrazolo[3,4-*b*:4',3'- *e*]pyridine.**

White solid, M.P. 208-210 °C, FT-IR (ATR) ῡ (cm^-1^):3496, 3259, 1607,1532, 1468, 1259, 1101, 802

^1^H NMR (400 MHz, DMSO-d_6_) /δ ppm: 11.30 (s, NH, 3H), 8.63 (s, OH, 1H), 8.52 (s, OH, 1H), 6.56 (d, *J*=8.0 Hz, 1H), 6.55 (s, 1H), 6.35 (d, *J*=8.0 Hz, 1H), 4.67 (s, 1H), 2.06 (s, 6H). C_15_H_15_N_5_O_2_, MS, m/z (%): 300.9 (M^+^), 272.1, 163.1, 110.1, 98, 81, 64.1, 41.1.


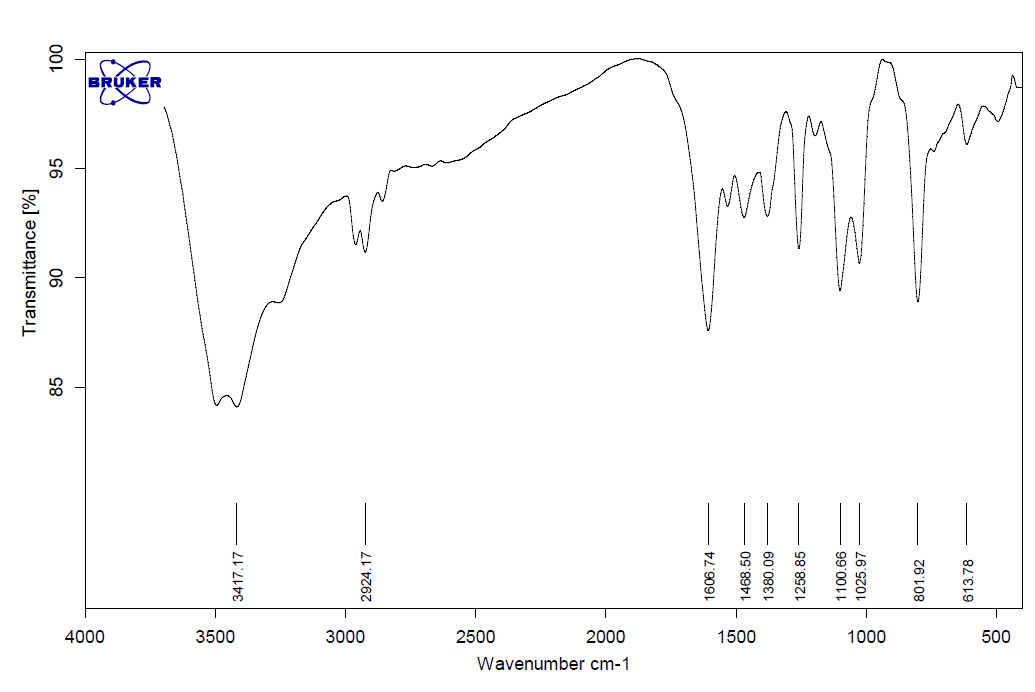


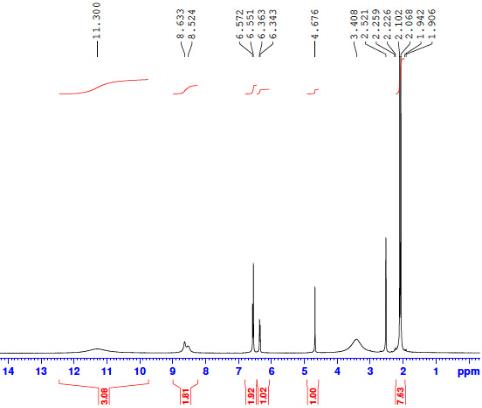


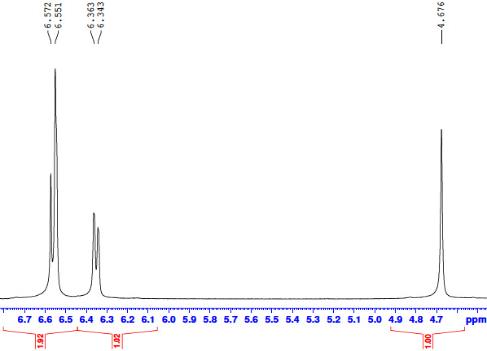


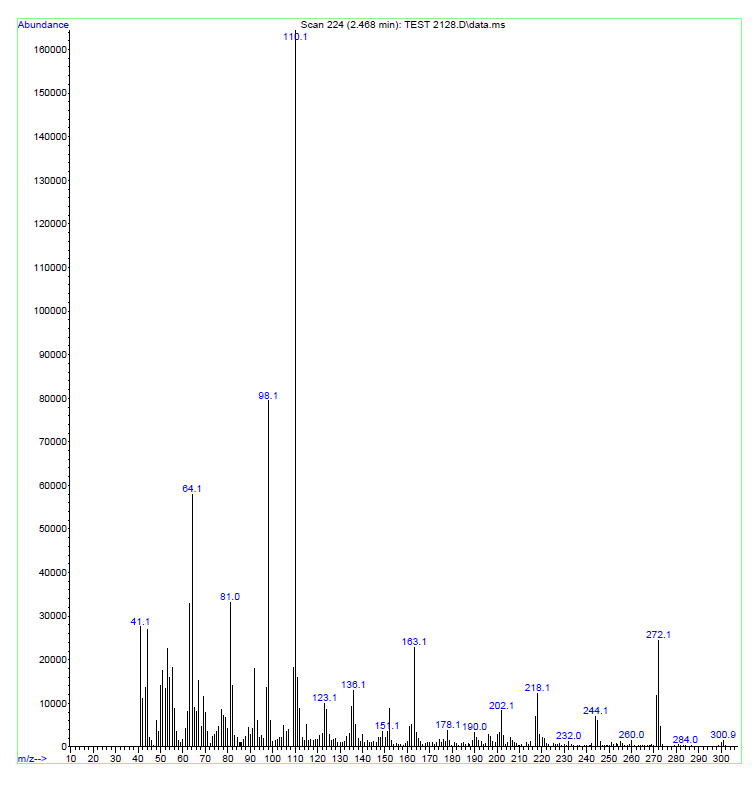


**4-(4-Bromo)-3,5-dimethyl-1,4,7,8-tetrahydrodipyrazolo[3,4-*b*:4',3'-*e*]pyridine**

White solid, M.P. 222-224 °C, FT-IR (ATR) ῡ (cm^-1^):3337, 2968, 1599, 1479, 1397, 831.

^1^H NMR (400 MHz, DMSO-d_6_) /δ ppm: 11.36 (s, NH, 3H), 7.15 (dd, *J*=8.0 Hz, *J*=5.6 Hz, 2H), 7.04 (t, *J*=8.8 Hz, 2H), 4.83 (s, 1H), 2.09 (s, 6H). C_15_H_14_BrN_5_, MS, m/z (%): 340, 339,338, (M^+^), 266, 264, 211, 185.1, 157, 155, 128.1, 109.1,89.1, 75.1,76, 50.1.


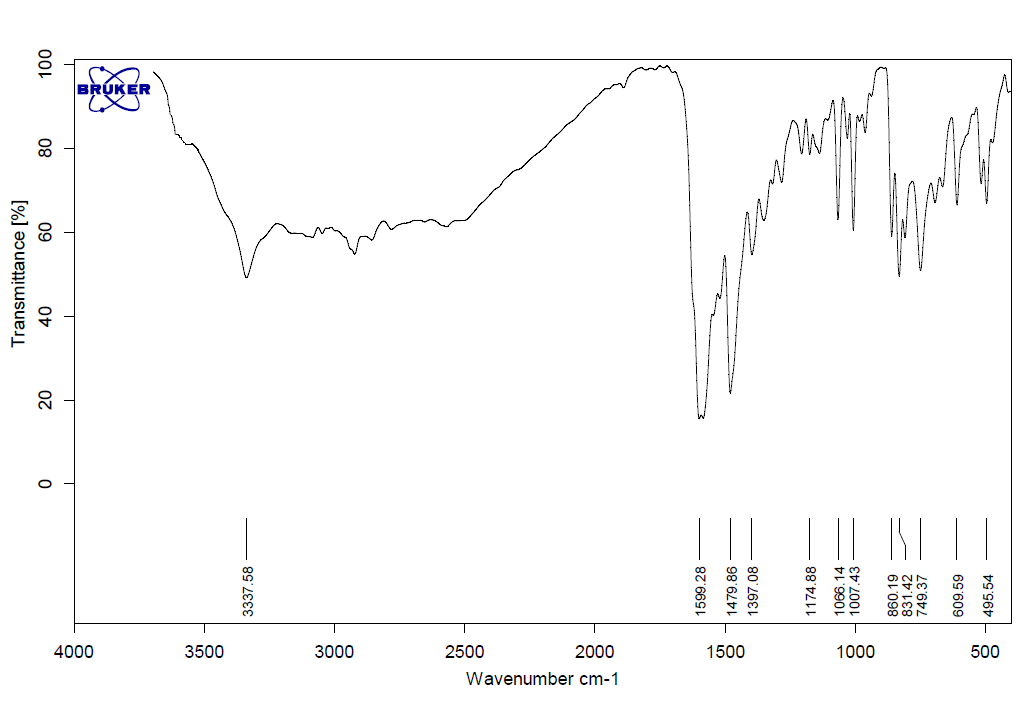


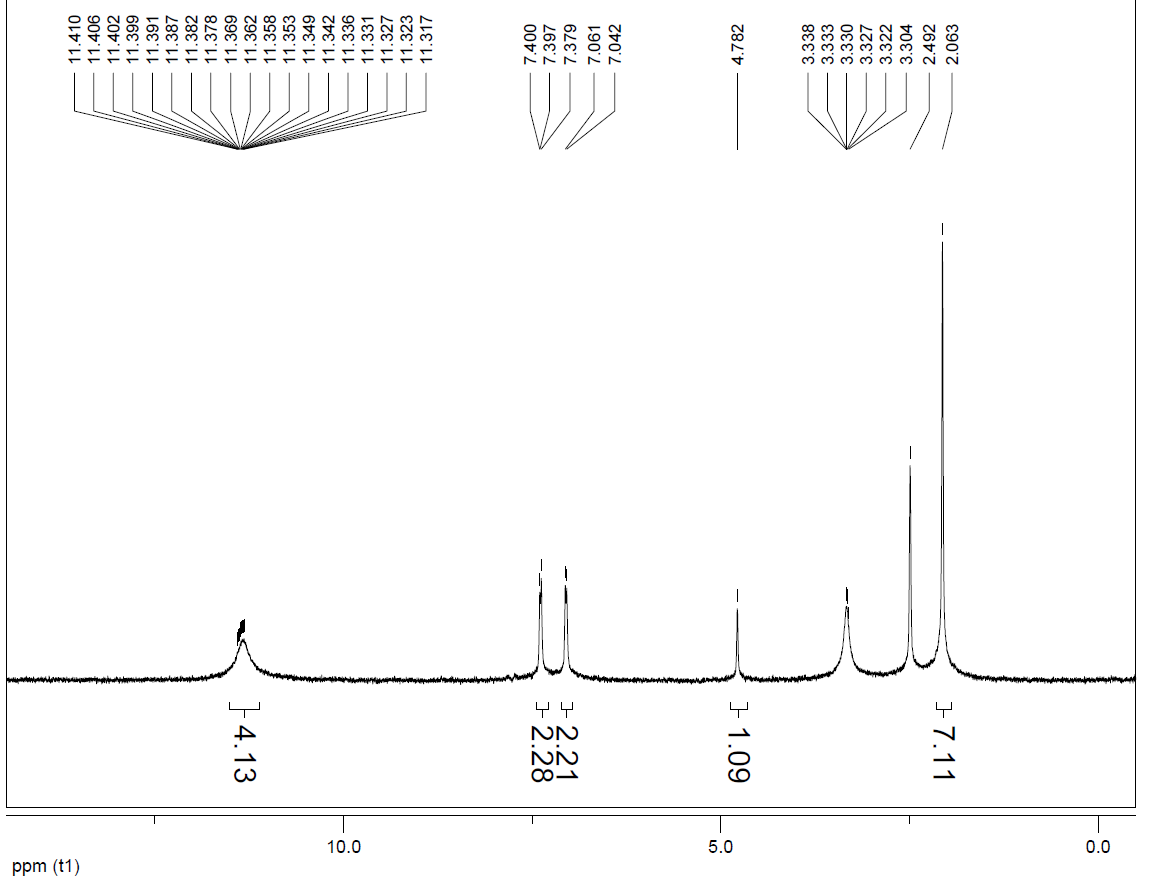

***
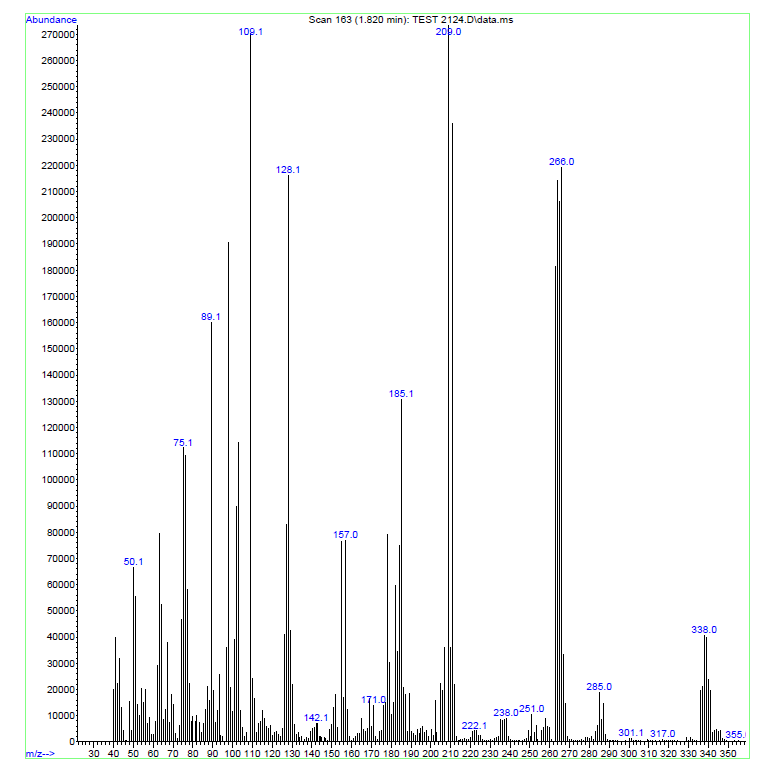
***

**3,5-Dimethyl-4-(4-dimethylaminophenyl)-1,4,7,8-tetrahydrodipyrazolo[3,4-b:4',3'-e]pyridine**

Yellow solid, M.P. 238-239 °C. FT-IR (ATR) ῡ (cm^-1^): 3423, 2915, 1604, 1365, 1171, 808. ^1^H NMR (400 MHz, DMSO-d_6_) /δ ppm: 11.24 (s, NH, 3H), 6.5 (d, *J*=8 Hz, 2H), 6.9 (d, *J*=8 Hz, 2H) 4.71 (s,1H), 3 (s, 3H), 2.8 (s, 3H), 2.5 (s, 3H), 2.07 (s, 3H). C_17_H_20_N_6_, MS m/z (%): 338 (M^+^), 266, 229.2, 228.1, 172.1, 156.1, 128.1, 98.1, 77.1, 64.1, 41.1.


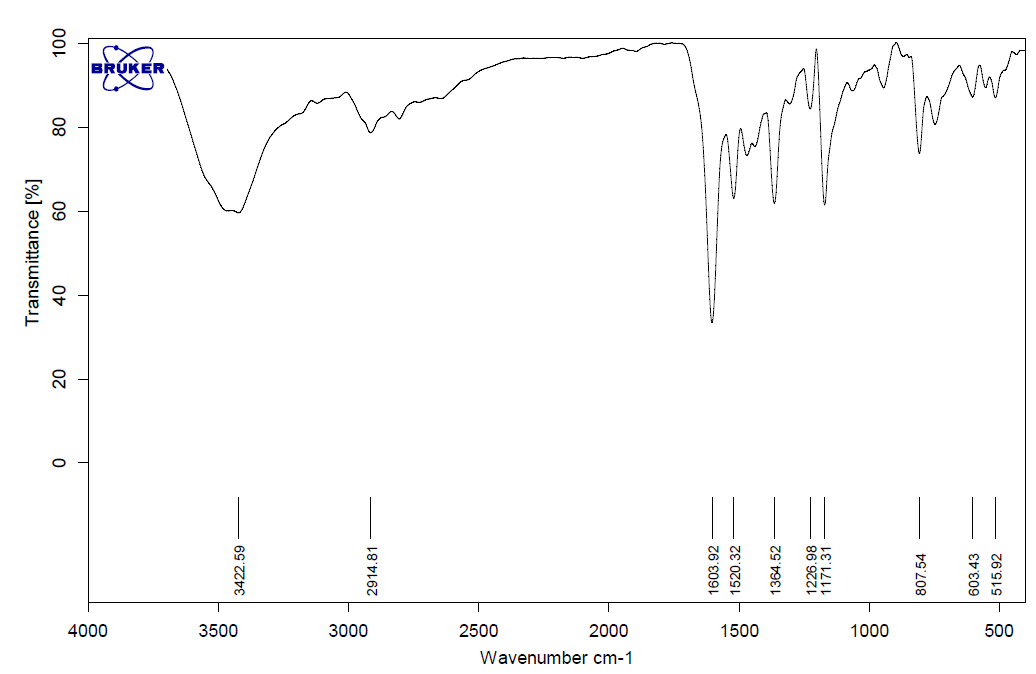

**
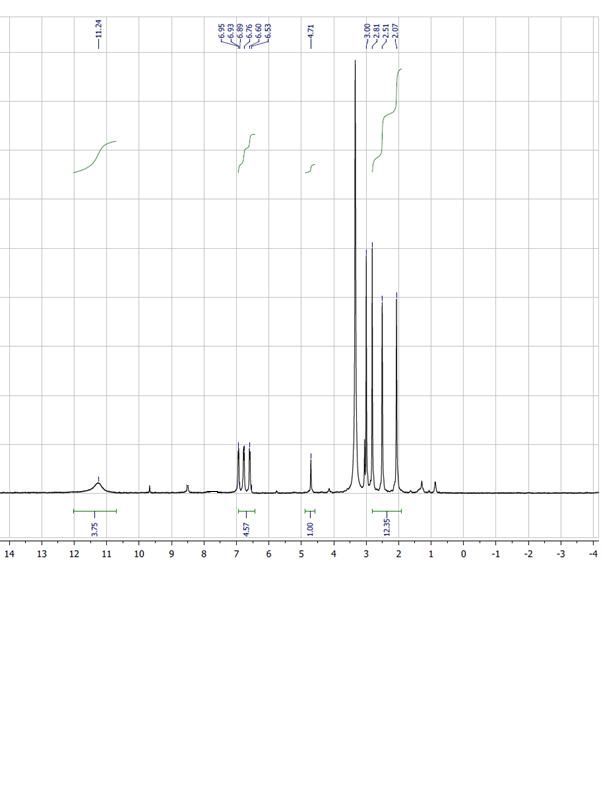
**

**
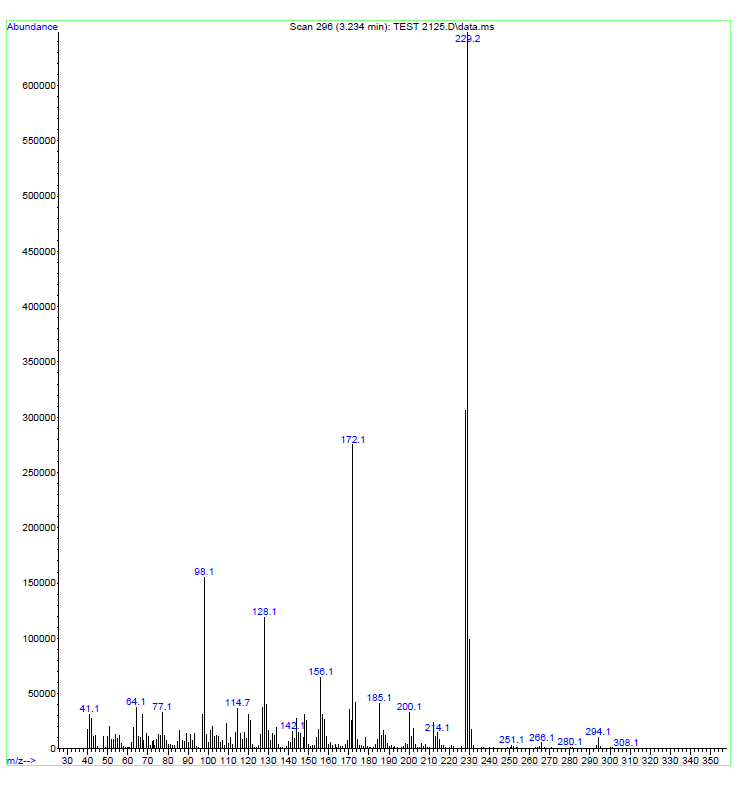
**

**1,4-Bis[(1,4,7,8-Tetrahydro-3,5-dimethyldipyrazolo[3,4-b:4',3'-e]pyridin-4-yl)] benzene**

light yellow solid, M.P. >300 °C. FT-IR (ATR) ῡ (cm^-1^): 3300, 1588, 1473, 1145, 732, 610. ^1^H NMR (400 MHz, DMSO-d_6_) /δ ppm: 10.99 (brs, 6H), 6.92 (s, 4H), 4.71(s, 2H), 1.99 (s, 12H). C_24_H_24_N_10_, MS, m/z (%):452 (M^+^) 338, 339, 340, 266, 264, 209, 211, 157, 155, 128, 109, 85, 44.


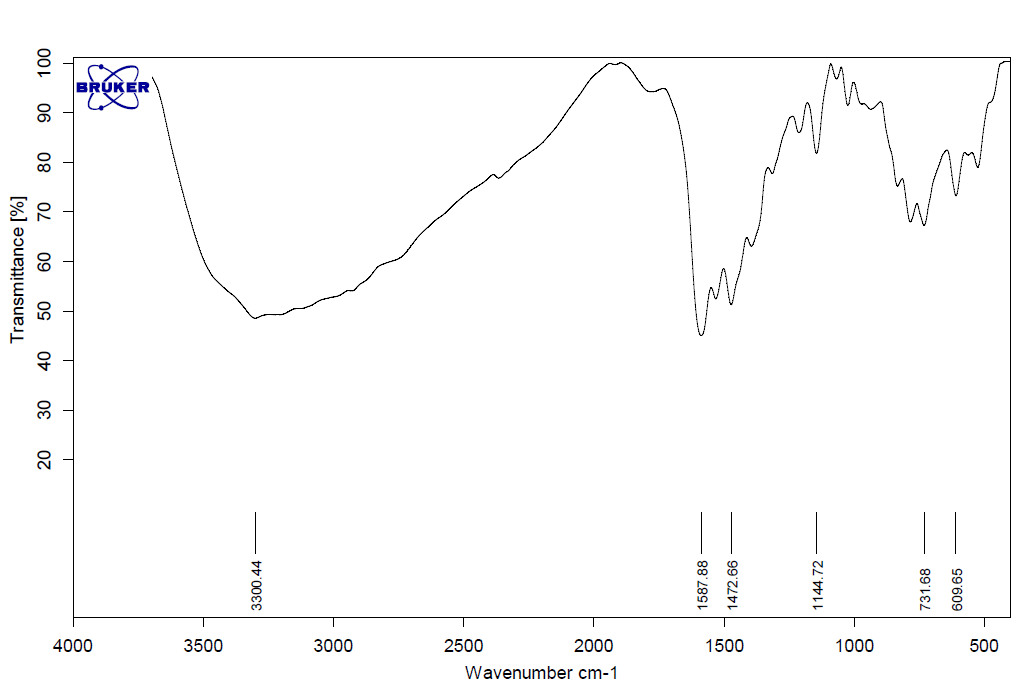


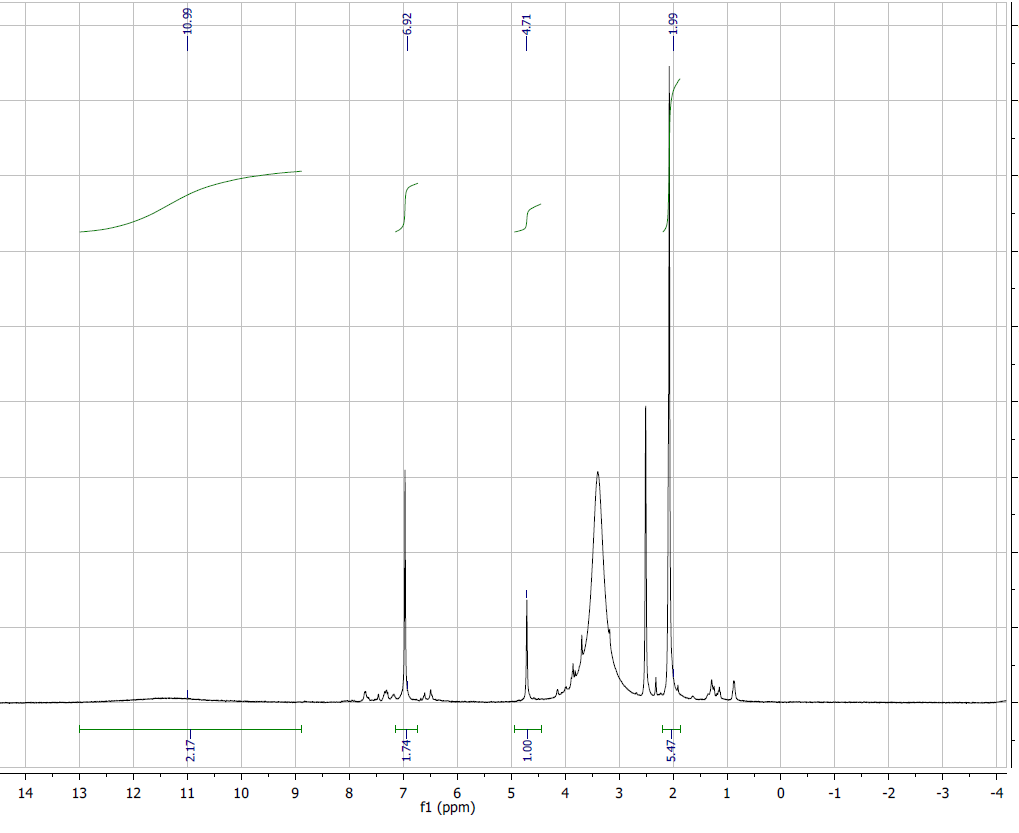


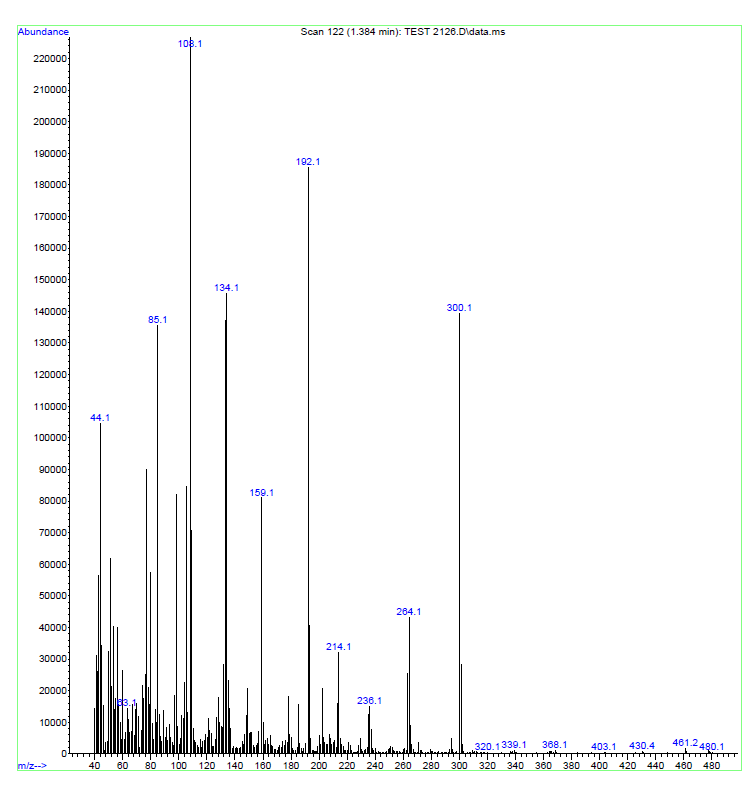


**4-(Z)-2,6-dimethylhepta-1,5-dien-1-yl)-3,5-dimethyl-*1,4,7,8-tetrahydrodipyrazolo[3,4-b:4',3'-e]pyridine.***

Orange solid, M.P. 250 °C. FT-IR (ATR) ῡ (cm^-1^): 3406, 1606, 1432, 1155, 766, 607. ^1^H NMR (400 MHz, DMSO-d_6_) /δ ppm: 10.66 (s, NH, 3H), 5.21 (s, 1H), 3.48-3.40 (m, 2H), 2.5 (s, 6H), 2.09 (s, 6H), 2.02-1.21 (m, 7 H).


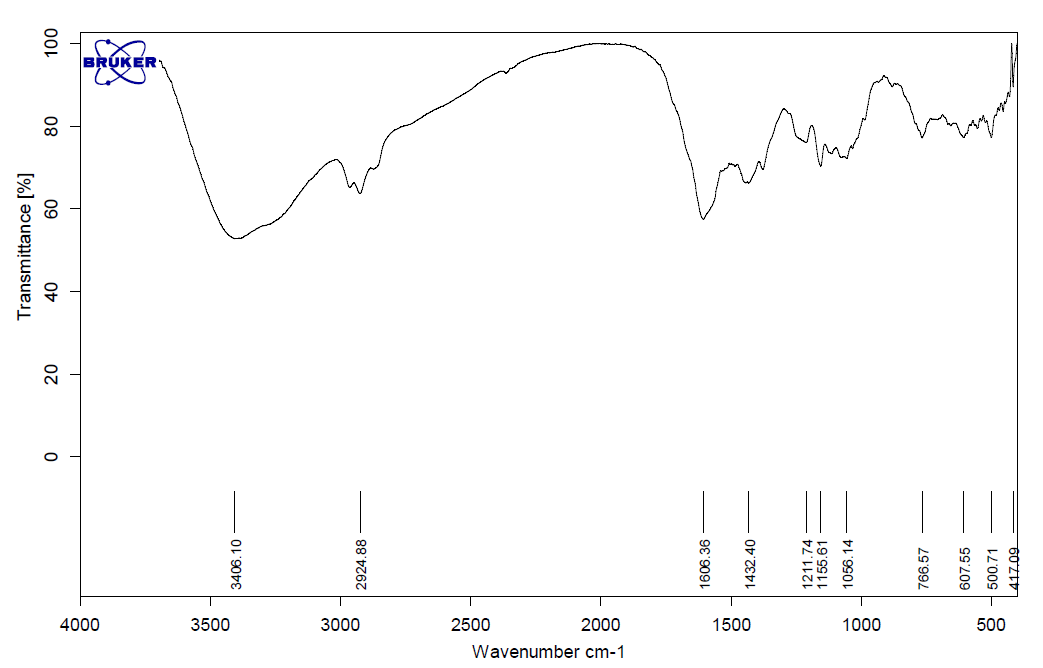


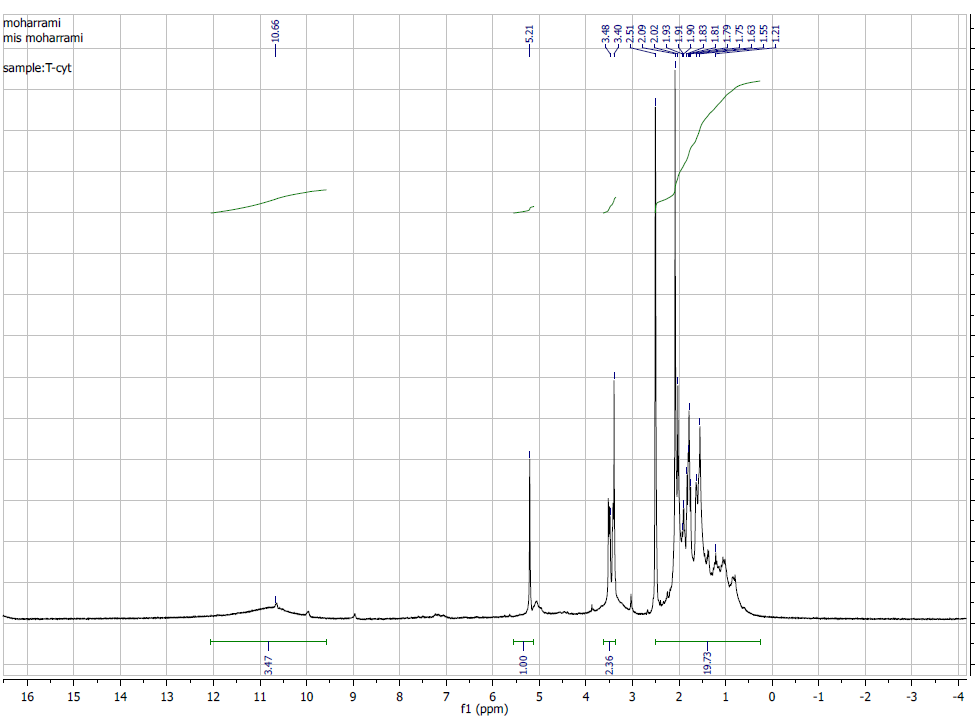


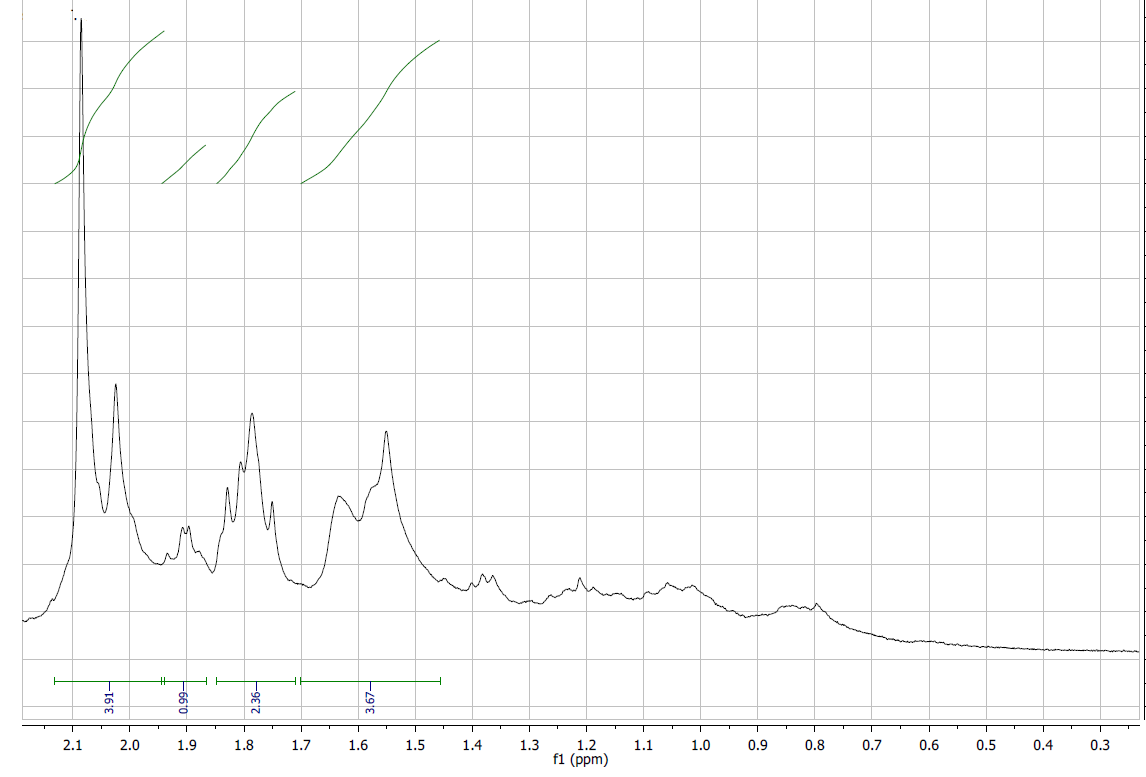

Supplement: Supplementary file 1 — Additional file 1: Spectroscopic data for the synthesized tetrahydrodipyrazolo[3,4-b:4′,3′-e] pyridine derivatives. [file 13065_2022_802_MOESM1_ESM.docx]
